# Postmenopausal levels of oestrogen, androgen, and SHBG and breast cancer: long-term results of a prospective study

**DOI:** 10.1038/sj.bjc.6601517

**Published:** 2004-01-06

**Authors:** A Zeleniuch-Jacquotte, R E Shore, K L Koenig, A Akhmedkhanov, Y Afanasyeva, I Kato, M Y Kim, S Rinaldi, R Kaaks, P Toniolo

**Affiliations:** 1 Department of Environmental Medicine, New York University School of Medicine, 650 First Avenue, New York, NY 10016, USA; 2 New York University Cancer Institute, New York University School of Medicine, New York, NY 10016, USA; 3 Department of Obstetrics and Gynecology, New York University School of Medicine, New York, 550 First Avenue NB9E2, New York, NY 10016, USA; 4 Department of Pathology, Karmanos Cancer Institute, Wayne State University, 110 E Warren Ave., Detroit, MI 48201, USA; 5 Department of Epidemiology and Population Health, Albert Einstein College of Medicine, Belfer Room 1303B, 1300 Morris Park Ave., Bronx, NY 10461, USA; 6 Hormones and Cancer Group, International Agency for Research on Cancer, 150 Cours Albert Thomas, 69372 Lyon, France

**Keywords:** oestrogen, androgen, oestradiol, oestrone, testosterone, androstenedione, DHEAS, SHBG, breast cancer

## Abstract

We assessed the association of sex hormone levels with breast cancer risk in a case–control study nested within the cohort of 7054 New York University (NYU) Women's Health Study participants who were postmenopausal at entry. The study includes 297 cases diagnosed between 6 months and 12.7 years after enrollment and 563 controls. Multivariate odds ratios (ORs) (95% confidence interval (CI)) for breast cancer for the highest quintile of each hormone and sex-hormone binding globulin (SHBG) relative to the lowest were as follows: 2.49 (1.47–4.21), *P*_trend_=0.003 for oestradiol; 3.24 (1.87–5.58), *P*_trend_<0.001 for oestrone; 2.37 (1.39–4.04), *P*_trend_=0.002 for testosterone; 2.07 (1.28–3.33), *P*_trend_<0.001 for androstenedione; 1.74 (1.05–2.89), *P*_trend_<0.001 for dehydroepiandrosterone sulphate (DHEAS); and 0.51 (0.31–0.82), *P*_trend_<0.001 for SHBG. Analyses limited to the 191 cases who had donated blood five to 12.7 years prior to diagnosis showed results in the same direction as overall analyses, although the tests for trend did not reach statistical significance for DHEAS and SHBG. The rates of change per year in hormone and SHBG levels, calculated for 95 cases and their matched controls who had given a second blood donation within 5 years of diagnosis, were of small magnitude and overall not different in cases and controls. The association of androgens with risk did not persist after adjustment for oestrone (1.08, 95% CI=0.92–1.26 for testosterone; 1.15, 95% CI=0.95–1.39 for androstenedione and 1.06, 95% CI=0.90–1.26 for DHEAS), the oestrogen most strongly associated with risk in our study. Our results support the hypothesis that the associations of circulating oestrogens with breast cancer risk are more likely due to an effect of circulating hormones on the development of cancer than to elevations induced by the tumour. They also suggest that the contribution of androgens to risk is largely through their role as substrates for oestrogen production.

Nine prospective studies have now reported on the association between endogenous sex hormone levels in postmenopausal women and subsequent breast cancer risk (Moore *et al*, 1986; Wysowski *et al*, 1987; Barrett-Connor *et al*, 1990; Gordon *et al*, 1990; Garland *et al*, 1992; Helzlsouer *et al*, 1994; Toniolo *et al*, 1995; Berrino *et al*, 1996; Dorgan *et al*, 1996; Thomas *et al*, 1997; Zeleniuch-Jacquotte *et al*, 1997; Hankinson *et al*, 1998; Cauley *et al*, 1999; Kabuto *et al*, 2000). The Endogenous Hormones and Breast Cancer Collaborative Group (TEHBCCG) conducted a pooled analysis of the original data of these studies and concluded that both oestrogen and androgen hormones were strongly associated with risk (TEHBCCG, 2002). Remaining questions include how long prior to diagnosis the associations between hormone levels and breast cancer are observed and whether androgens play a part independent of their role as substrates for oestrogen production. The New York University (NYU) Women's Health Study was one of the first prospective studies to report a positive association between oestrogens and androgens and breast cancer risk (Toniolo *et al*, 1995). We expand here our initial results that were based on 130 cases for oestrogen analyses and 85 cases for androgen analyses. The present report includes 297 cases diagnosed between 6 months and 12.7 years after enrollment in the study. This study has nearly twice as many cases as any previously published cohort study. Owing to the large sample size and extended follow-up, we were able to assess the association of breast cancer risk with hormone levels in serum samples collected five or more years prior to diagnosis. To explore whether the presence of a growing cancer results in an increase in circulating hormone levels, we also examined the rate of change per year in hormone and sex-hormone binding globulin (SHBG) levels in 95 cases and their matched controls who contributed a second blood donation within 5 years of diagnosis.

## MATERIALS AND METHODS

### The NYU Women's Health Study cohort

Between 1985 and 1991, the NYU Women's Health Study enrolled 14 275 healthy women aged 34–65 years at the Guttman Breast Diagnostic Institute, a breast cancer screening centre in New York City (Toniolo *et al*, 1991,^1995^). Women who had been pregnant or taken hormonal medications in the 6 months preceding their visit were not eligible. Women were classified as postmenopausal if they reported no menstrual cycles in the previous 6 months, a total bilateral oophorectomy, or a hysterectomy without total oophorectomy prior to natural menopause and their age was 52 years or older. A total of 7054 participants (49.4%) were postmenopausal at the time of initial blood donation. After written informed consent was obtained, demographic, medical, anthropometric, reproductive, and dietary data were collected through self-administered questionnaires. Nonfasting peripheral venous blood (30 ml) was drawn prior to breast examination. After centrifugation, serum samples were divided into 1 ml aliquots and immediately stored at −80°C for subsequent biochemical analyses. Up to 1991, women who returned for annual breast cancer screening were invited to contribute additional blood donations.

### Nested case–control study of breast cancer

Breast cancer cases were identified through active follow-up of the cohort by mailed questionnaires approximately every 2–4 years and telephone interviews for nonrespondents, as well as record linkage with state cancer registries in New York, New Jersey, and with the US National Death Index. A capture–recapture analysis estimated the ascertainment rate in our cohort to be 95% (Kato *et al*, 1999). Only incident cases (i.e. diagnosed at least 6 months after blood donation) of invasive breast cancer were included to avoid selection bias from ‘prevalent’ cases. Medical and pathology reports were requested to confirm the diagnoses.

For each case, two controls were selected at random from the appropriate risk sets. The risk set for a case consisted of all women postmenopausal at enrollment who were alive and free of cancer at the time of diagnosis of the case and who matched the case on age at entry (±6 months), date of enrollment (±3 months), and number and dates (±6 months) of subsequent blood donations, if any. Menopausal status was confirmed by measuring follicle-stimulating hormone (FSH) in all women for whom the lagtime between last menstrual period and blood donation was less than 2 years and all women who were less than 60 years old at entry and reported having had a hysterectomy without complete bilateral oophorectomy and women with FSH levels ⩽12.75 mIU ml^−1^ were excluded.

### Laboratory analyses

All assays were conducted in the Hormones and Cancer Group at the International Agency for Research on Cancer in Lyon, France. Assays were selected based on the results of a validity study (Rinaldi *et al*, 2001). Members of a matched set were always analysed in the same batch. Oestradiol, oestrone, androstenedione, and FSH were measured by direct double-antibody radioimmunoassays from DSL (Diagnostic System Laboratories, TX, USA), testosterone and dehydroepiandrosterone sulphate (DHEAS) were measured by direct radioimmunoassays from Immunotech (Marseille, France) and SHBG was measured by a direct ‘sandwich’ immunoradiometric assay (Cis-Bio, Gif-sur-Yvette, France). The mean intra- and interbatch coefficients of variation were 4.9 and 13.2% respectively for oestradiol (at a concentration of 257 pmol l^−1^), 6.7 and 14.4% for oestrone (at 74 pmol l^−1^), and 7.8 and 13.5% for androstenedione (at 1.4 nmol l^−1^), 8.7 and 15.8% for testosterone (at 1.4 nmol l^−1^), 5.4 and 14.7% for DHEAS (at 1.62 *μ*mol l^−1^), and 5.6 and 13.5% for SHBG (at 40 nmol l^−1^).

### Statistical methods

The distributions of known risk factors in cases and controls were compared using the conditional logistic regression model, to take into account the matching (Breslow and Day, 1980). To test for differences in hormone (and SHBG) levels between case and control subjects, we used a mixed-effects regression model taking into account the matched design: after logarithmic transformation to reduce departures from the normal distribution, the hormone (or SHBG) levels were modelled as a function of a random stratum effect (matched set) and a fixed effect for case–control status (Cnaan *et al*, 1997).

To compute odds ratios (ORs), serum measurements were categorised into quintiles, using the frequency distribution of the cases and the controls combined. The matched set data were analysed using conditional logistic regression (Breslow and Day, 1980). Odds ratios were computed relative to the lowest quintile. Reported trend test *P*-values correspond to hormone variables treated as ordered categorical variables. Analyses were also performed on log-transformed continuous variables. The log_2_-transformation was used because it leads to the OR associated with a doubling in hormone level, an estimate more easily interpretable than those obtained from other logarithmic transformations (TEHBCCG, 2002). All *P*-values are two-sided.

To explore whether the presence of a growing cancer results in an increase in circulating levels of sex hormones, we calculated the rate of change per year in hormone and SHBG levels in the subset of subjects for whom two blood donations were available and the second blood donation was within 5 years of the index date. We compared values in cases and their matched controls using a mixed-effects regression model. These analyses were controlled for age through the matching. We also controlled for baseline level of hormone (or SHBG), rate of change per year in body mass index, and time since menopause.

## RESULTS

By 1 March 1998, the start date of the latest round of follow-up, 306 participants postmenopausal at enrollment were first diagnosed with invasive adenocarcinoma of the breast 6 months or more after entry into the study. Nine cases (3%) were excluded for the following reasons: postmenopausal status not confirmed by FSH analysis (two cases), lack of serum (two cases), both selected controls developed cancer (breast or other) and their serum was reserved for analyses in which they were the index cases (five cases). The remaining 297 cases are included in the present analysis. Pathology reports were obtained for 232 cases (78%), and 51 additional cases (16%) were confirmed by the Tumour Registries. Among the 594 controls initially selected, 31 (5%) were excluded for the following reasons: postmenopausal status not confirmed by FSH analysis (four controls), participant had been selected as a control for a previous case (seven controls), participant developed breast (six controls) or another cancer (14 controls), and her serum was reserved for analyses in which she was the index case.

Table 1

**Table 1:** Selected characteristics of study subjects, NYU Women's Health Study, 1985–1998

	**Case subjects (*n*=297)**	**Control subjects (*n*=563)**
Age (years) at enrollment, median (10th–90th percentiles)	60 (54–64)	60 (54–64)
Age (years) at diagnosis, median (10th–90th percentiles)	66.1 (58.5–72.5)	
Age (years) at menarche, median (10th–90th percentiles)	12 (11–14)	13 (11–15)
Nulliparous* (%)	77 (30.9%)	117 (23.4%)
Age (years) at first full-term pregnancy, median (10th–90th percentiles)	25 (20 –31)	24.5 (20–30)
Age (years) at menopause, median (10th–90th percentiles)	50 (41–55)	50 (42–55)
*First-degree family history of breast cancer*		
No	234 (78.8%)	445 (79.1%)
Yes, one affected relative ⩾45 years old	43 (14.5%)	93 (16.5%)
Yes, one affected relative <45 years old or more than one affected relative	20 (6.7%)	25 (4.4%)
		
Prior breast biopsy** (%)	83 (27.9%)	121 (21.5%)
Prior bilateral oophorectomy (%)	47 (15.8%)	78 (13.8%)
Height (cm), median (10th–90th percentiles)	163 (155–170)	162 (152–170)
Weight (kg)***, median (10th–90th percentiles)	68.1 (54.0–84.9)	63.6 (53.6–81.7)
Body mass index (kg m^−2^)***, median (10th–90th percentiles)	25.7 (21.0–31.4)	24.3 (20.8–30.9)

* *P*<0.10,

** *P*=0.05,

*** *P*<0.01.

presents selected characteristics of participants. The median age at enrollment was 60 years (range, 44–65) and the median age at diagnosis was 66.1 years (range, 52.6–77.4). Compared to controls, the case subjects were characterised by a higher frequency of nulliparity (31 *vs* 23%, *P*=0.09), a higher frequency of history of breast biopsy (28 *vs* 22%, *P*=0.05), a higher median weight (68.1 *vs* 63.6 kg, *P*=0.002), and higher median body mass index (25.7 *vs* 24.3 kg m^−2^, *P*=0.007).

Table 2

**Table 2:** Median (10th and 90th percentiles) serum levels of hormones and SHBG in case and matched control subjects

**Hormone (unit)**	**Case subjects (*n*=297)**	**Control subjects (*n*=563)**	***P*-value^a^**
Oestradiol (pmol l^−1^)	88.86 (57.04–150.40)	81.78 (54.04–137.76)	0.005
Oestrone (pmol l^−1^)	104.58 (68.46–175.20)	99.03 (60.20–153.51)	<0.001
Testosterone (nmol l^−1^)	0.87 (0.31–1.89)	0.79 (0.21–1.64)	0.002
Androstenedione (nmol l^−1^)	2.70 (1.23–5.90)	2.45 (0.96–4.71)	<0.001
DHEAS (*μ*mol l^−1^)	2.37 (0.92–6.39)	2.13 (0.71–5.07)	0.002
SHBG (nmol l^−1^)	42.51 (22.1–76.3)	48.11 (24.0–89.6)	<0.001

a *P*-values are from the mixed-effects regression model on log_2_-transformed variables, controlling for matching factors.

presents descriptive statistics on the hormone and SHBG levels. The median levels of all oestrogen and androgen hormones were 5% (oestrone) to 10% (DHEAS) higher among cases than controls. These differences were all highly statistically significant. SHBG was 13% higher in controls than in cases (*P*<0.001).

Table 3

**Table 3:** ORs (95% CI) for breast cancer by quintiles of serum sex hormone and SHBG levels among postmenopausal women in the NYU Women's Health Study

	**Quintiles**					
**Hormone**	**1**	**2**	**3**	**4**	**5**	***P* for trend**
*Oestradiol (pmol l*^−*1*^*)*						
Cutpoints	<62.87	62.87–77.18	77.19–92.48	92.49–116.34	>116.34	
#cases/#controls	47/123	61/110	52/119	62/108	72/98	
Model a^a^	1.0	1.56 (0.95–2.56)	1.14 (0.69–1.89)	1.63 (0.98–2.71)	2.33 (1.40–3.88)	0.004
Model b^b^	1.0	1.63 (0.98–2.69)	1.16 (0.69–1.92)	1.69 (1.00–2.84)	2.49 (1.47–4.21)	0.003
Model c^c^	1.0	1.52 (0.91–2.53)	1.08 (0.64–1.80)	1.50 (0.88–2.55)	2.06 (1.18–3.60)	0.04

*Oestrone (pmol* *l*^−*1*^*)*						
Cutpoints	<74.43	74.43–93.02	93.03–109.93	109.94–133.61	>133.61	
#cases/#controls	40/132	64/108	59/113	62/110	72/99	
Model a^a^	1.0	2.26 (1.36–3.78)	2.12 (1.24–3.62)	2.35 (1.38–4.02)	2.97 (1.76–5.02)	<0.001
Model b^b^	1.0	2.44 (1.43–4.16)	2.30 (1.32–4.00)	2.46 (1.41–4.28)	3.24 (1.87–5.58)	<0.001
Model c^c^	1.0	2.32 (1.36–3.95)	2.08 (1.18–3.66)	2.15 (1.22–3.80)	2.67 (1.50–4.76)	0.006

*Testosterone (nmol l*^−*1*^*)*						
Cutpoints	<0.42	0.42–0.66	0.67–0.94	0.95–1.39	>1.39	
#cases/#controls	47/125	60/112	58/114	64/108	68/103	
Model a^a^	1.0	1.63 (0.99–2.68)	1.51 (0.92–2.48)	1.84 (1.11–3.02)	2.15 (1.29–3.59)	0.005
Model b^b^	1.0	1.69 (1.01–2.81)	1.57 (0.94–2.61)	1.93 (1.16–3.23)	2.37 (1.39–4.04)	0.002
Model c^c^	1.0	1.64 (0.98–2.74)	1.50 (0.90–2.50)	1.87 (1.12–3.13)	2.05 (1.19–3.53)	0.01

*Androstenedione (nmol* *l*^−*1*^*)*						
Cutpoints	<1.43	1.43–2.16	2.17–2.90	2.91–3.91	>3.91	
#cases/#controls	48/123	60/112	54/117	60/111	74/97	
Model a^a^	1.0	1.31 (0.82–2.10)	1.17 (0.73–1.90)	1.44 (0.89–2.33)	2.04 (1.28–3.25)	0.006
Model b^b^	1.0	1.29 (0.80–2.10)	1.20 (0.74–1.97)	1.45 (0.89–2.37)	2.07 (1.28–3.33)	<0.001
Model c^c^	1.0	1.24 (0.77–2.03)	1.15 (0.70–1.88)	1.43 (0.87–2.34)	1.89 (1.16–3.07)	<0.001

*DHEAS (μmol* *l*^−*1*^*)*						
Cutpoints	<1.15	1.15–1.83	1.84–2.63	2.64–3.97	>3.97	
#cases/#controls	50/120	56/115	56/114	64/107	69/101	
Model a^a^	1.0	1.21 (0.76–1.94)	1.28 (0.79–2.08)	1.53 (0.95–2.47)	1.84 (1.12–3.03)	0.02
Model b^b^	1.0	1.08 (0.66–1.74)	1.30 (0.80–2.13)	1.44 (0.88–2.34)	1.74 (1.05–2.89)	<0.001
Model c^c^	1.0	1.08 (0.66–1.75)	1.23 (0.75–2.01)	1.39 (0.85–2.27)	1.61 (0.97–2.69)	<0.001

*SHBG (nmol* *l*^−*1*^*)*						
Cutpoints	<29.70	29.70–40.39	40.40–52.31	52.32–67.85	>67.85	
#cases/#controls	74/98	64/108	63/109	49/123	47/125	
Model a^a^	1.0	0.81 (0.52–1.26)	0.73 (0.46–1.17)	0.49 (0.31–0.78)	0.50 (0.31–0.81)	<0.001
Model b^b^	1.0	0.77 (0.49–1.22)	0.73 (0.45–1.17)	0.46 (0.28–0.75)	0.51 (0.31–0.82)	<0.001
Model c^c^	1.0	0.82 (0.51–1.30)	0.78 (0.48–1.28)	0.52 (0.31–0.87)	0.58 (0.34–0.98)	0.01

a Controlling for matching factors only.

b Adjusting for age at menarche (continuous), family history of breast cancer (no, one affected first-degree relative >45 years old, one affected first-degree relative <45 years old or more than one affected first-degree relative), parity/age at first birth (nulliparous, ⩽20 years at first full-term pregnancy, 21–25 years at first full-term pregnancy, 26–30 years at first full-term pregnancy, >30 years at first full-term pregnancy), history of total oophorectomy, and history of breast biopsy.

c Adjusting for all variables in model b, plus body mass index (ln) and height (ln).

presents ORs for breast cancer by quintile of hormone and SHBG levels. Significant trends of increasing risk with increasing levels of all hormones were observed in matched analyses not adjusted for additional factors (model a). As body mass index is a determinant of circulating oestrogen levels (Vermeulen and Verdonck, 1979; Poortman *et al*, 1981; Kaye *et al*, 1991) and therefore on the same causal pathway as oestrogens, we present adjusted analyses both including and excluding this variable (plus height, as recommended by Michels *et al*, 1998). Adjusting for age at menarche, parity, age at first birth, family history of breast cancer, and history of breast biopsy, one variable at a time (data not shown) or simultaneously (Table 3, model b) did not materially affect the ORs. Adjusting for BMI and height (model c) led to a reduction in ORs, which was more pronounced for oestrogens than for androgens: The top quintile ORs associated with oestradiol and oestrone were reduced by 17 and 18%, respectively, whereas for androgens the ORs were reduced by 7–13%. All associations remained strongly significant. The strongest association with breast cancer was observed for oestrone with a 3.2-fold increase in risk for women in the highest quintile relative to the lowest in model b (the corresponding OR was 2.7 in model c). A strong inverse association was observed with SHBG, with a 49% reduction in risk for women in the top quintile, as compared to women in the lowest quintile.

Table 4

**Table 4:** ORs (95% CI) for breast cancer by quintiles of serum sex hormone and SHBG levels among postmenopausal women in the NYU Women's Health Study with 5 or more years between blood donation and index date

	**Quintiles**					
**Hormone**	**1**	**2**	**3**	**4**	**5**	***P* for trend**
*Oestradiol (pmol* *l*^−*1*^*)*						
Cutpoints	<62.87	62.87–77.18	77.19–92.48	92.49–116.34	>116.34	
#cases/#controls	31/75	32/69	35/77	37/77	53/70	
Model a^a^	1.0	1.09 (0.59–2.01)	1.04 (0.58–1.86)	1.08 (0.58–2.00)	2.03 (1.11–3.71)	0.04
Model b^b^	1.0	1.08 (0.58–2.03)	1.00 (0.55–1.82)	1.13 (0.60–2.13)	2.18 (1.16–4.09)	0.001

*Oestrone (pmol l*^−*1*^*)*						
Cutpoints	<74.43	74.43–93.02	93.03–109.93	109.94–133.61	>133.61	
#cases/#controls	28/77	45/77	35/79	41/70	42/66	
Model a^a^	1.0	1.78 (0.96–3.30)	1.39 (0.71–2.73)	1.92 (0.99–3.72)	2.07 (1.08–3.96)	0.05
Model b^b^	1.0	1.90 (1.00–3.60)	1.45 (0.72–2.91)	2.03 (1.02–4.06)	2.28 (1.17–4.47)	0.04

*Testosterone (nmol* *l*^−*1*^*)*						
Cutpoints	<0.42	0.42–0.66	0.67–0.94	0.95–1.39	>1.39	
#cases/#controls	36/88	36/75	39/73	41/64	39/68	
Model a^a^	1.0	1.36 (0.75–2.45)	1.45 (0.81–2.61)	1.75 (0.97–3.16)	1.71 (0.93–3.15)	0.06
Model b^b^	1.0	1.29 (0.70–2.36)	1.44 (0.79–2.63)	1.70 (0.92–3.13)	1.85 (0.99–3.48)	0.02

*Androstenedione (nmol* *l*^−*1*^*)*						
Cutpoints	<1.43	1.43–2.16	2.17–2.90	2.91–3.91	>3.91	
#cases/#controls	33/83	42/70	34/72	34/73	47/68	
Model a^a^	1.0	1.49 (0.82–2.69)	1.19 (0.65–2.18)	1.26 (0.68–2.33)	1.81 (1.03–3.19)	0.1
Model b^b^	1.0	1.47 (0.80–2.69)	1.17 (0.63–2.17)	1.27 (0.68–2.36)	1.88 (1.05–3.35)	0.004

*DHEAS (μmol* *l*^−*1*^*)*						
Cutpoints	<1.15	1.15–1.83	1.84–2.63	2.64–3.97	>3.97	
#cases/#controls	33/75	39/76	39/80	37/66	41/66	
Model a^a^	1.0	1.20 (0.68–2.13)	1.19 (0.66–2.13)	1.34 (0.74–2.43)	1.57 (0.84–2.92)	0.18
Model b^b^	1.0	1.10 (0.61–1.97)	1.15 (0.64–2.08)	1.30 (0.71–2.39)	1.48 (0.78–2.79)	0.19

*SHBG (nmol* *l*^−*1*^*)*						
Cutpoints	<29.70	29.70–40.39	40.40–52.31	52.32–67.85	>67.85	
#cases/#controls	45/65	35/80	40/78	36/77	35/69	
Model a^a^	1.0	0.59 (0.33–1.06)	0.67 (0.38–1.19)	0.60 (0.34–1.06)	0.68 (0.38–1.22)	0.25
Model b^b^	1.0	0.58 (0.32–1.06)	0.68 (0.37–1.22)	0.60 (0.34–1.08)	0.70 (0.39–1.26)	0.31

a Controlling for matching factors only.

b Adjusting for age at menarche (continuous), family history of breast cancer (no, one affected first-degree relative >45 years old, one affected first-degree relative <45 years old or more than one affected first-degree relative), parity/age at first birth (nulliparous, ⩽20 years at first full-term pregnancy, 21–25 years at first full-term pregnancy, 26–30 years at first full-term pregnancy, >30 years at first full-term pregnancy), history of total oophorectomy, and history of breast biopsy.

presents ORs from analyses limited to the 191 cases, whose blood was drawn 5 or more years before diagnosis, and their matched controls. Results were in the same direction for all the hormones and SHBG as in the analyses including all cases, although the ORs tended to be closer to unity and the trends were no longer significant for DHEAS and SHBG.

A second blood sample collected within 5 years of the index date was available for cases and controls from 95 matched sets. The mean duration between first and second blood donations was 31 months (s.d., 17.4 months), and the mean duration between second blood donation and diagnosis was 28 months (s.d., 19.8 months). Table 5

**Table 5:** Mean rates of change per year in hormone and SHBG levels in 95 cases and 172 matched controls who had a second preindex date measurement within 5 years of diagnosis

	**Mean rates of change^a^ per year**					
**Hormone**	**Crude**			**Adjusted^b^**		
	**Cases**	**Controls**	***P*-value**	**Cases**	**Controls**	***P*-value**
Oestradiol (pmol l^−1^)	−0.005	−0.015	0.71	0.002	−0.018	0.45
Oestrone (pmol l^−1^)	0.007	−0.013	0.33	0.008	−0.015	0.28
Testosterone (nmol l^−1^)	0.045	0.048	0.97	0.069	0.030	0.50
Androstenedione (nmol l^−1^)	0.080	−0.017	0.09	0.104	−0.034	0.01
DHEAS (*μ*mol l^−1^)	−0.018	−0.012	0.82	−0.015	−0.012	0.91
SHBG (nmol l^−1^)	0.001	−0.043	0.14	−0.011	−0.035	0.41

a Rate of change=[log_2_ (hormone at second visit)−log_2_ (hormone at first visit)]/[number of years between visits].

b Adjusted for log_2_ baseline level, rate of change in body mass index and time since menopause.

reports the mean rates of change per year in hormone and SHBG levels, separately for cases and controls. For SHBG and all hormones except androstenedione, the mean changes per year were of very small amplitude and the matched analysis showed no differences between cases and controls. For androstenedione, the mean rate of change suggested a slight increase in levels over time (i.e. with decreasing time to diagnosis) among cases, whereas there was a slight decrease among controls. This marginally statistically significant difference (*P*=0.09) became significant after adjusting for baseline androstenedione level, rate of change in body mass index and time since menopause (*P*=0.01).

Table 6

**Table 6:** ORs for breast cancer associated with a doubling in androgen and SHBG levels, with and without adjustment for oestrogen levels

		**Adjusted for**	
	**Unadjusted**	**Oestradiol**	**Oestrone**
*Testosterone*			
OR (95% CI)	1.23 (1.08–1.41)	1.17 (1.00–1.36)	1.08 (0.92–1.26)
*P*-value	0.001	0.04	0.31

*Androstenedione*			
OR (95% CI)	1.33 (1.13–1.57)	1.26 (1.05–1.52)	1.15 (0.95–1.39)
*P*-value	<0.001	0.01	0.15

*DHEAS*			
OR (95% CI)	1.23 (1.07–1.42)	1.16 (0.99–1.36)	1.06 (0.90–1.26)
*P*-value	0.004	0.06	0.47

presents the ORs associated with a doubling in androgen levels with and without adjustment for oestrogen levels. In unadjusted analyses, the ORs varied from 1.23 (testosterone and DHEAS) to 1.33 (androstenedione) and were all highly statistically significant. The ORs decreased slightly, but remained statistically significant (or close to, for DHEAS) after adjustment for oestradiol. The ORs, though, became close to one (1.06–1.15) and no longer statistically significant after adjustment for oestrone.

## DISCUSSION

In 1995, we reported a positive association between endogenous oestrogens and breast cancer risk based on the first 130 cases observed among the postmenopausal participants in the NYU Women's Health Study (Toniolo *et al*, 1995). With an additional 7 years of follow-up, we confirm our initial results that increasing circulating levels of oestradiol and oestrone are associated with increasing risk of breast cancer. We also confirm the positive association with testosterone levels observed previously (Zeleniuch-Jacquotte *et al*, 1997). These results are in agreement with those of most published prospective studies (TEHBCCG, 2002).

Our initial results on total oestradiol and oestrone levels (Toniolo *et al*, 1995) were questioned (Kuller, 1995) because measurements were carried out using commercial radioimmunoassay kits and oestradiol levels were higher than oestrone levels, which was contrary to expectation in postmenopausal women. To address this criticism, and prior to performing the assays reported here, we carried out a validity study to assess various oestrogen and androgen assays (Rinaldi *et al*, 2001), as recommended by Hankinson *et al* (1994). This study allowed us to select direct assays with high intrabatch reproducibility, high correlation with indirect assays, and accurate ranking of subjects by hormone serum concentrations. We reassayed all the sera from the previous study using these methods. In the pooled analysis of nine prospective studies, no differences in endogenous oestrogen- and androgen-associated relative risks between studies that had used a method incorporating a purification step and studies that had used a direct, no-extraction method were found.

Oestrone, with an OR of 3.24 (95% confidence interval (CI)=1.87–5.58; model b) for women in the top quintile, appeared more strongly associated with risk than oestradiol (OR=2.49; 95% CI=1.47–4.21). This result is consistent with the results of the pooled analysis of prospective studies where the OR for women in the top quintile of oestradiol was 2.00 (95% CI=1.47–2.71) and for women in the top quintiles of oestradiol and oestrone were 2.19 (95% CI=1.48–3.22), respectively (TEHBCCG, 2002). As oestradiol has greater potency and binds with oestrogen receptor-*α* with greater affinity than oestrone (Zava *et al*, 1997), a stronger association of risk with oestradiol than with oestrone is usually expected. Measurement error is not likely to be responsible for our finding the reverse because the attenuation of the ORs so caused is inversely related to the reliability of the measurements. In our study, oestrone had a slightly lower reliability (intraclass correlation coefficient (ICC)=0.58) than oestradiol (ICC=0.66), so that the ORs observed with oestrone would be expected to be more attenuated than with oestradiol. New evidence points to a role of oestrogens in the development of breast cancer independent of oestrogen receptor mediation, through metabolites such as 4- and 16*α*-hydroxyoestrogens (Yager, 2000). The stronger association of risk with oestrone than with oestradiol could therefore result from the higher concentrations of oestrone metabolites, themselves resulting from the higher concentrations of oestrone than oestradiol observed in postmenopausal women. Finally, it has been argued that a stronger association would be observed with the fraction of oestradiol not bound to SHBG, because it is readily available to breast cells, than with total oestradiol, as we (Toniolo *et al*, 1995) and others (TEHBCCG, 2002) have previously found. But because we did not measure the various oestrogen fractions in this study we could not assess this possibility.

Whereas oestrogens are known to directly stimulate breast cell proliferation, it is not clear whether the role of androgens is only as precursors of oestrogens, or whether they have a direct role in breast cancer development through conversion into oestrogens in the breast itself or by direct stimulation of the growth and division of breast cells. Multivariate analysis, which is mostly used to control for confounding, may also be used to assess underlying mechanisms (Szklo and Nieto, 1999): If an association of androgens with breast cancer risk persisted after adjusting for oestrogens, it would indicate that androgens may act through more direct mechanisms in addition to increasing oestrogen levels. In our study, the association of androgens with risk persisted after adjustment for oestradiol, but not after adjustment for oestrone, the oestrogen that was most strongly associated with risk in these data (Table 6). These results suggest that the contribution of androgens to breast cancer risk is largely through their role as substrates for oestrogen production. These analyses, though, did not take into account the error in measurement resulting from using a single serum sample to quantify a woman's long-term average hormone levels. We attempted to correct our estimates for such measurement error (Kim and Zeleniuch-Jacquotte, 1997), using the repeated measurement data from the 317 controls who had contributed two blood donations. The reliability correlation coefficients estimated from these data were: 0.66 (95% CI=0.61–0.70) for oestradiol, 0.58 (95% CI=0.53–0.63) for oestrone, 0.63 (95% CI=0.58–0.67) for testosterone, 0.64 (95% CI=0.59–0.68) for androstenedione, and 0.92 (95% CI=0.91–0.93) for DHEAS, respectively. Our attempts to correct for measurement error, though, led to uninterpretable results because of the instability in the corrected estimates resulting from the multicollinearity among hormone variables: among controls, the Spearman's correlation coefficients for testosterone, androstenedione, and DHEAS with oestradiol were 0.47, 0.43, and 0.42, respectively, and with oestrone were 0.57, 0.51, and 0.57 respectively.

The oestradiol-adjusted ORs associated with a doubling of androgen levels that we observed were very similar to those observed in the pooled analysis of prospective studies (TEHBCCG, 2002), that is, 1.27 for androstenedione (*vs* 1.26 in our study), 1.15 for DHEAS (*vs* 1.16), and 1.32 for testosterone (*vs* 1.17). Oestrone-adjusted ORs were not presented in that study. Whether the contribution of androgens to breast cancer risk is direct or indirect, it would be of interest to identify the source of elevated levels of androgens. The increased DHEAS serum concentrations in women who develop breast cancer suggest increased adrenal androgen secretion. In a prospective study including 53 postmenopausal cases, Dorgan *et al* (2001) assessed the androstenedione : 11*β*-hydroxyandrostenedione ratio, which is depressed when the adrenals are the primary source of androstenedione but elevated when the ovaries are the primary source. They concluded that both the adrenals and the ovaries appear to contribute to elevated androstenedione levels in postmenopausal women. Further research on the factors that contribute to elevated androgen levels is warranted.

The weak association of breast cancer risk with DHEAS that we observed in our initial analysis (Zeleniuch-Jacquotte *et al*, 1997) became stronger with the increased sample size. A substantial association was also observed in several prospective studies and in the pooled analysis of these studies. The risk for women in the highest quintile of DHEAS was 60% higher than for women in the lowest quintile. DHEAS can be converted into DHEA, itself convertible to androstenedione. DHEAS can also be converted into 5-androstenediol, which in postmenopausal women has oestrogenic properties through binding to oestrogen receptors (Seymour-Munn and Adams, 1983). As pointed out previously, these results should caution against the use of DHEA as a supplement with various ‘antiageing’ properties (Hankinson *et al*, 1998). DHEA oral supplementation leads to significant increases in circulating levels of DHEAS, testosterone, androstenedione, oestrone, and oestradiol (Genazzani *et al*, 2001).

Adjusting for body mass index resulted in an attenuation of the ORs associated with oestrogen levels. These results were expected in the light of the role of adipose tissue in producing oestrogens in postmenopausal women. Spearman's correlation coefficients of body mass index with oestradiol and oestrone were 0.39 and 0.34, respectively. Although smaller, an attenuation of the ORs associated with androgen levels was also observed. A positive association of obesity with increased levels of testosterone has been reported (TEHBCCG, 2003). Obesity could therefore contribute to breast cancer by increasing the amount of androgens available for conversion to oestrogens, in addition to increasing their rate of conversion. The weak positive correlations between body mass index and androgen levels that we observed: 0.13, 0.17, and 0.09 with testosterone, androstenedione, and DHEAS, respectively, are consistent with such an effect.

To further investigate whether elevated levels of sex hormones are involved in the induction of breast cancer and not simply a byproduct of the tumour, we conducted an analysis limited to the 191 cases who had donated blood 5 or more years prior to diagnosis. This analysis showed results in the same directions as overall analyses, although the tests for trend did not reach statistical significance for DHEAS and SHBG. Our study is the first to assess the association of breast cancer risk with circulating levels of sex hormones measured well before diagnosis in a fairly large group of cases. The results suggest that the observed associations are more likely due to an effect of circulating hormones on the development of clinical cancer than to an increase in circulating hormone levels induced by the tumour.

The availability of a second measurement within 5 years of the index date for cases and controls from 95 matched sets allowed us to examine changes in the levels of hormones and SHBG prior to the index date. If the elevated levels of hormones observed in case subjects were due to the presence of tumours, then these levels would be expected to increase at a faster rate in cases (as they approach diagnosis) than in controls. The mean rate of change of androstenedione indicated a slight increase in serum levels in cases but not in controls. However, for all other hormones and SHBG, changes were of negligible amplitude and not significantly different in cases and controls. These results suggest that the presence of the growing tumour does not have a major effect on circulating levels of sex hormones or SHBG.

For completeness and to maximise statistical power, we included in the present analysis all the case subjects in our previous report if diagnosed after 6 months in the study, namely, 82 cases (28% of the total) and their controls included in our initial report were reassayed and included in this report. Excluding these subjects did not materially affect the results (data not shown).

In conclusion, our results show that the associations between oestrogen and androgen levels and breast cancer risk are present 5 or more years prior to diagnosis and therefore more likely represent an effect of circulating hormones than of the tumour. This is a key finding towards establishing that sex hormones are causally related to breast cancer. Our results also suggest that the contribution of androgens to breast cancer risk is largely through their role as substrates for oestrogen production.
